# Prolonged Antibiotic Exposure During Gestation Increases the Severity of Perinatal Asphyxia as Measured by EEG Reactivity in Rodents

**DOI:** 10.3390/neurolint18050092

**Published:** 2026-05-15

**Authors:** Vlad-Petru Morozan, Mihai Stancu, Mara Ioana Ionescu, Ana-Maria Catrina, Alexandra Mocanu, Vladimir Suhăianu, Andrei-Vladimir Iacovache, Ana-Teodora Chirilă, Andrei Bordeianu, Leon Zăgrean, Ana-Maria Zăgrean, Mihai Moldovan

**Affiliations:** 1 Division of Physiology—Neuroscience, Carol Davila University of Medicine and Pharmacy, 050474 Bucharest, Romania; vlad-petru.morozan@drd.umfcd.ro (V.-P.M.); mihai.stancu@umfcd.ro (M.S.); mara-ioana.ionescu@umfcd.ro (M.I.I.); andrei-vladimir.iacovache25@rez.umfcd.ro (A.-V.I.); ana-teodora.chirila0721@stud.umfcd.ro (A.-T.C.); andrei.bordeianu@stud.umfcd.ro (A.B.); leon.zagrean@umfcd.ro (L.Z.); ana-maria.zagrean@umfcd.ro (A.-M.Z.); 2 Department of Plastic and Reconstructive Microsurgery, Dr. Carol Davila Central Military Emergency University Hospital, 010825 Bucharest, Romania; 3 Division of Neurobiology, Faculty of Biology, Ludwig-Maximilians-Universität München, 82152 Planegg-Martinsried, Germany; 4 Department of Pediatrics II, Marie Curie Emergency Children’s Hospital, 077120 Bucharest, Romania; 5 Cantacuzino National Military Medical Institute for Research and Development, 077035 Cernica, Romania; catrina.ana-maria@cantacuzino.ro (A.-M.C.); suhaianu.vladimir@cantacuzino.ro (V.S.); 6 Doctoral School of Philosophy, Faculty of Philosophy, University of Bucharest, 060024 Bucharest, Romania; alexandra.mocanu2@s.unibuc.ro; 7 Department of Obstetrics and Gynecology, Filantropia Clinical Hospital, Carol Davila University of Medicine and Pharmacy, 011132 Bucharest, Romania; 8 Department of Neuroscience, University of Copenhagen, 2200 Copenhagen, Denmark; 9 Department of Clinical Neurophysiology, Rigshospitalet, 2100 Copenhagen, Denmark; 10 Department of Neurology, North Zealand Hospital, 3400 Hillerød, Denmark

**Keywords:** burst suppression, hypoxic–ischemic brain injury, EEG reactivity, burst count, photic stimulation, neurodevelopment, perinatal brain injury, maternal antibiotics

## Abstract

Background/Objectives: Birth asphyxia is a frequent neonatal complication in humans. Its outcome is variable, and the factors underlying this variability remain incompletely understood. Maternal gut microbiome impairment has been proposed as one factor that may influence offspring neurodevelopment, especially when the immature brain is exposed to additional vulnerability such as perinatal asphyxia (PA). Building on our previous maternal microbiome disruption model and on our prior observation that electroencephalography (EEG) reactivity to photic stimulation under deep anesthesia detects functional impairment two months after PA, we assessed whether this reactivity was further impaired after prolonged gestational antibiotic administration and whether probiotics modulated this effect. Methods: Wistar dams received antibiotics, probiotics, antibiotics with probiotics, or control treatment, and offspring underwent PA. Adult EEG reactivity to photic stimulation was assessed during chloral hydrate-induced burst suppression. Burst count reactivity (BCR) was used as the primary event-based readout of stimulus-evoked burst recruitment and was compared with the suppression-ratio-based burst-suppression reactivity index (BSRi). Results: Burst suppression remained reactive to photic stimulation in all groups. BCR was lower after gestational antibiotic treatment than in controls. The magnitude of the effect was attenuated by probiotics coadministration. BSRi showed the same overall pattern. Conclusions: Prolonged gestational antibiotic exposure increased the severity of perinatal asphyxia as measured by EEG reactivity in the adult offspring. The converging BCR and BSRi results support burst-suppression reactivity as a functional neurophysiological readout in this PA model and support further methodological development of EEG reactivity measures for translational studies of hypoxic–ischemic brain injury.

## 1. Introduction

The outcome of birth asphyxia, a frequent neonatal complication in humans, is notoriously variable. Some neonates recover with limited detectable sequelae, whereas others develop persistent neurological impairment, encompassing the hypoxic–ischemic brain injury (HIBI) [1]. The factors that underlie this variability remain poorly understood and are being explored in experimental perinatal asphyxia (PA) models [1,2,3].

Maternal factors are increasingly recognized as potential modifiers of PA outcome. Dietary interventions, maternal metabolic state, and maternal inflammatory signaling can influence the vulnerability of the immature brain to HIBI [4,5,6,7]. In this context, maternal gut microbiome impairment has garnered attention for its potential role in influencing neurodevelopmental outcomes in offspring, especially in situations that increase brain vulnerability, such as PA [8,9].

We developed an experimental rat model to study the impact of maternal gut microbiome disruption on PA [9]. We found that antibiotic exposure amplified the HIBI following PA. The same work also refined the antibiotic cocktail to reduce pregnancy-related adverse effects while retaining maternal gut microbiome disruption, thereby providing a practical model for subsequent long-term outcome studies [9]. Nevertheless, our studies were primarily focused on short-term outcome measures, including acute seizure burden, hippocampal injury, neuroinflammation, and early behavioral reflexes [9,10].

Electroencephalography (EEG) can provide insight into persistent network-level dysfunction in hypoxic–ischemic encephalopathy. Traditional EEG outcome measures focus on assessing seizure burden [11,12] and background activity [13]. More recently, increasing attention has been directed toward methods that assess the changes in background EEG activity in response to a controlled stimulation, referred to as EEG reactivity [14,15]. We translated these changes into a rat model to assess the reactivity to photic stimulation under anesthesia [16]. The reactivity measure was sensitive enough to detect functional impairment two months after PA.

Building upon our previous maternal microbiome disruption model and our prior finding that EEG reactivity to photic stimulation under deep anesthesia detects functional impairment two months after PA [9,16], we investigated whether this reactivity was further impaired after prolonged gestational antibiotic administration. For assessing the microbiome modulation, we did not attempt to duplicate our previous studies, but rather, we tested the potential modulatory effect of probiotic administration [17,18,19,20].

We tested EEG reactivity to photic stimulation under deep anesthesia, where the EEG consists of high-amplitude bursts alternating with periods of suppression, referred to as the burst-suppression (BS) patterns [21,22,23]. The suppression ratio (SR), which measures the fraction of time spent in suppression, increases with anesthetic depth and is widely used as a quantitative descriptor of BS [24,25]. Nevertheless, during BS, external stimuli can trigger bursts, providing a functional readout of residual network responsiveness that has been shown in both experimental and clinical settings [14,26,27,28].

In our previous translational EEG study, reactivity was assessed as the decrease in SR during stimulation, normalized by the SR before stimulation, and was referred to as the burst-suppression reactivity index (BSRi) [16]. BSRi is useful because it is based on a continuous time-domain measure and is standardized to pre-stimulation anesthetic depth. However, BSRi cannot distinguish whether stimulation-related decreases in SR are explained mainly by an increased number of bursts, by longer burst duration, or by both. This distinction is important because a sensory stimulus during BS is expected to act primarily by increasing the probability of burst recruitment, whereas SR integrates both burst occurrence and burst duration.

To gain further mechanistic support, the current study focused on burst counting as an event-based readout of stimulus-evoked burst recruitment. Burst counting is rule-based [21] and methodologically more challenging than BSRi. We therefore used manually supervised burst counting to assess the burst count reactivity (BCR) as the increase in the number of bursts during stimulation. This assessment was controlled by inter-rater validation and by comparison with BSRi derived from the reviewer-supervised automated workflow.

## 2. Materials and Methods

### 2.1. Experimental Design

**Figure 1:**
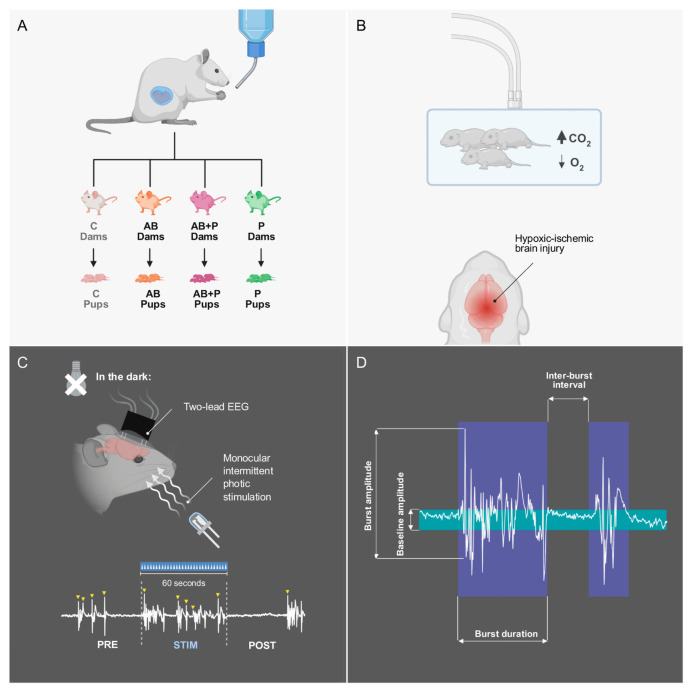
**Experimental design and EEG reactivity protocol.** Schematic overview of the study workflow. (**A**): Pregnant dams were assigned to four gestational treatment groups: control (C), antibiotics (AB), antibiotics plus probiotics (AB+P), and probiotics (P). Their offspring underwent the corresponding prenatal exposure condition. (**B**): On postnatal day 6, pups were subjected to perinatal asphyxia using a hypoxic hypercapnic gas mixture, as an experimental model of hypoxic–ischemic brain injury. (**C**): At two months postnatal, EEG recordings were performed under chloral hydrate-induced burst suppression using a two-lead epidural montage. Reactivity to monocular intermittent photic stimulation was assessed across consecutive 60 s pre-stimulation (PRE), stimulation (STIM), and post-stimulation (POST) epochs. (**D**): A representative burst description, indicating burst amplitude, baseline amplitude, burst duration and inter-burst interval (suppression). Figure created in BioRender. Racoviță, A. (2026) https://BioRender.com/y2pklsr (accessed on 7 April 2026).

Pregnant Wistar rats were assigned to four experimental treatment groups: antibiotics (AB), probiotics (P), antibiotics combined with probiotics (AB+P), and control (C). Their offspring underwent a perinatal asphyxia (PA) protocol (Figure 1A). Neurophysiological investigations were carried out at postnatal two months. EEG reactivity studies were performed under deep general anesthesia at the burst-suppression level. The final analysis included 45 rats in total, distributed as follows: 9 AB-PA, 13 P-PA, 12 AB+P-PA, and 11 C-PA. These 45 rats were derived from 20 dams (5 per group). Overall, 51.1% of the rats were male (23/45). The percentage of males in each group was 66.7% in AB-PA (6/9), 46.2% in P-PA (6/13), 50.0% in AB+P-PA (6/12), and 45.5% in C-PA (5/11). Sex information is provided to document that the dataset was relatively balanced by sex across groups. Because the study was not powered for sex-stratified inference, sex was not included in the primary statistical models [29]. Following completion of the experiments, the rats were euthanized by cervical dislocation under deep general anesthesia.

All experimental procedures were approved by the Local Committee for Animal Research, Carol Davila University of Medicine and Pharmacy, Bucharest, Romania (7845/25.03.2021), and were conducted in accordance with Directive 2010/63/EU on the protection of animals used for scientific purposes. The project was authorized by the Ilfov Directorate for Veterinary Health and Food Safety (project authorization no. 37/23.08.2023) and received approval from the Ethics Committee of the Cantacuzino National Military Medical Institute for Research and Development (CE 319/2023).

### 2.2. Maternal Treatment

Starting on gestational day 11, corresponding to early neurodevelopmental patterning in the rat [30], dams in the antibiotic (AB) and antibiotic + probiotic (AB+P) groups received a broad-spectrum antibiotic cocktail in the drinking water throughout the rest of the gestation period. This regimen was selected based on prior studies in which similar protocols have been shown to induce profound perturbations of the maternal gut microbiota [31,32,33,34]. The final formulation consisted of ampicillin (1 mg/mL), vancomycin (0.5 mg/mL), neomycin (5 mg/mL), and meropenem (1 mg/mL) [9].

Probiotic treatment began on the first day of gestation in the probiotic (P) and antibiotic + probiotic (AB+P) groups. Dams received a multi-strain probiotic preparation (Vivomixx, Mendes, Lugano, Switzerland), containing *Lactobacillus* spp., *Bifidobacterium* spp., and *Streptococcus thermophilus*, diluted in the drinking water at 6.1 × 10^8^ colony-forming units (CFU)/mL. To avoid simultaneous exposure of the probiotic to antibiotics, treatments in the antibiotic + probiotic (AB+P) group were temporally separated following a previously established protocol [35]: antibiotics were administered overnight (16:00–08:00), whereas the probiotic was provided during the remaining hours of the light cycle. Both treatments continued daily until birth, with drinking water replaced every 24 h.

Maternal water intake and body weight were monitored throughout gestation as general indicators of treatment tolerability.

### 2.3. Perinatal Asphyxia

On postnatal day 6, pups from each of the four groups underwent perinatal asphyxia (PA). The asphyxia gas (9% O_2_, 20% CO_2_ in N_2_) flowed through an open non-rebreathing system at a rate of 2 L/min for 90 min (Figure 1B). Arterial oxygen saturation, respiratory rate, and heart rate were monitored using a MouseOx pulse oximeter (Starr Life Sciences Corp., Oakmont, PA, USA), and body temperature was maintained at 37 °C by adjusting a heating pad based on a surface thermistor [10].

### 2.4. EEG Electrodes and Surgical Implantation

Anesthesia was induced with 3.0% *v*/*v* isoflurane in a sealed chamber [36], followed by an intraperitoneal injection of ketamine 60 mg/kg (Bioveta, Cluj-Napoca, Romania) and xylazine 5 mg/kg (Bioveta, Cluj-Napoca, Romania). To maintain anesthesia throughout the procedure, supplemental intramuscular ketamine (60 mg/kg) was administered as required. Surgical depth was ensured by the absence of the tail pinch response.

After the rat was secured in a stereotaxic frame (Neurostar, Tübingen, Germany), the cranial fur was removed using a cordless electric clipper, and the exposed skin was disinfected with Betadine. The scalp was then vertically incised, and the skin and soft tissues were removed to uncover the cranium. Using a cotton swab, FeCl_3_ was applied to maximize the surface adherence of the dental cement.

Custom epidural electrode arrays were constructed using standard 2.54 mm pitch pin header connectors to serve as the primary headstage. Bare silver wires were soldered directly to the connector pins to form individual cortical leads. To provide physical durability and electrical insulation across the wire shafts, the soldered assemblies were coated with acrylic resin, intentionally leaving only the distal tips exposed. The exposed silver tips that would come in contact with the brain were electrochemically chlorinated in an aqueous HCl solution using a 9 V direct current source. This step deposits a stable surface layer that minimizes polarization and ensures reliable signal transduction at the cortical interface.

Electrode holes were drilled into the cranium at the following coordinates relative to the bregma: 5 mm anterior, +/−2.35 mm lateral; 6 mm posterior, +/−3.35 mm lateral [37]. The electrodes were placed in contact with the dura mater, and the connector was secured to the skull with dental cement (UNIFAST Trad, GC Dental Products Corp., Kasugai, Japan). Postoperative care included 0.5 mL of intraperitoneal saline for hydration, after which the animals recovered in their home cages for two days prior to EEG recordings.

### 2.5. EEG Recordings

We recorded cortical EEG from two epidural leads, referred to hereafter as EEG. Following isoflurane induction as described in Section 2.4, chloral hydrate was administered intraperitoneally at 0.4 g/kg body weight, a dose previously sufficient to induce discontinuous EEG patterns [37]. If burst suppression, visually assessed as suppression occupying more than 50% of the epoch, was not achieved, up to two supplemental injections of 0.1 g/kg chloral hydrate were administered. Recordings were stopped when the EEG recovered toward a nearly continuous pattern.

Recordings were acquired using a BIOPAC MP150 system with EEG100C amplifier modules (BIOPAC Systems, Inc., Goleta, CA, USA) configured at a sample rate of 1000 Hz, a 10 dB gain, and a 1 to 35 Hz hardware bandpass filter. The implanted head stage was connected to the acquisition system, and a ground electrode was attached at the base of the tail via 1% lithium recording gel (OTE Biomedica, Genoa, Italy). A 2-channel fronto-parietal bipolar EEG montage was recorded with AcqKnowledge 4.2 (BIOPAC Systems, Inc., Goleta, CA, USA) [16]. Stimulation voltage was recorded in parallel with the EEG. Cardiac activity was monitored throughout by electrocardiography (ECG).

### 2.6. Photic Stimulation and Data Preprocessing

Visual stimulation was delivered via a custom script developed in MATLAB (version 2022b, MathWorks, Inc., Natick, MA, USA). Stimulations were carried out in 60 s epochs, interlaced by 60 s recovery intervals. A similar paradigm has been used in human studies of deep anesthetic coma to assess burst-suppression reactivity [14,28] and continuous EEG macrostate reactivity [15].

Intermittent photic stimulation was carried out via a light-emitting diode placed in front of the right eye at a frequency of 0.5 Hz. The left eye was covered. For the entire duration of stimulation, the rats were placed in darkness to ensure constant lighting. The resulting two EEG channels were acquired as ipsilateral and contralateral with respect to the stimulated side. The raw files were converted to the structured Hierarchical Data Format version 5 (HDF5) format. EEG signals were preprocessed using a zero-phase 4th-order Butterworth bandpass filter (1–35 Hz).

All recording files were anonymized, and a de-identification key was kept separately for downstream analysis.

### 2.7. Burst Annotation, Semi-Automated Burst-Suppression Analysis, and Metric Extraction

A custom Python v3.13.9-based GUI tool (BURST—Burst-sUppression Reviewer-Supervised analysis Tool) was developed to support reviewer-supervised, semi-automated analysis of burst-suppression EEG recordings on a trial-by-trial basis. The software imported preprocessed EEG recordings, reconstructed stimulus triggers, derived a rectified-smoothed EEG envelope, generated candidate burst intervals using threshold- and duration-based rules, and allowed manual validation and correction of burst annotations. For each valid trial, the software exported epoch-specific burst counts, suppression-rate summaries, and epoch durations, which were then used for downstream derivation of burst count reactivity and suppression-ratio-based indices.

Stimulus triggers were reconstructed directly from the stimulation channel by detecting rising-edge events after thresholding relative to the stimulation peak. For each trial, a rectified EEG envelope was generated from the primary EEG channel by full-wave rectification followed by temporal smoothing. In the relative-threshold workflow, the burst-detection threshold was computed from the rectified signal using a noise-reference estimate, which was derived from sliding-window minima and then scaled by a threshold factor.

The automated layer classified the rectified-smoothed EEG signal relative to the trial-specific threshold and assembled a binary burst-suppression representation. This was then cleaned iteratively using minimum burst-duration and minimum suppression-duration constraints, and generated candidate burst intervals with defined onset and offset times. For the used analysis, the default temporal constraints were a minimum burst duration of 0.45 s, a minimum suppression duration of 0.50 s, and a fixed amplitude threshold of four times the baseline (Figure 1D), inspired by the clinical critical care EEG standards, adapted for use with rodent epidural recordings [21]. Automatically proposed bursts were then reviewed by the investigator and could be accepted, edited, added, deleted, or repositioned before final trial validation.

**Figure 2:**
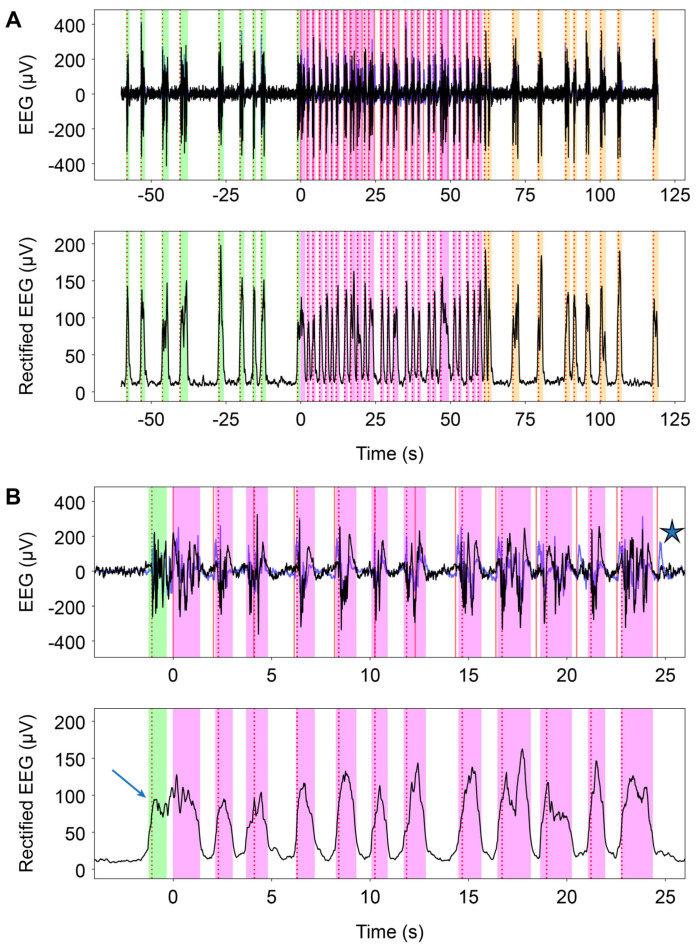
**Representative trial from the P-PA group illustrating manual and automatic burst identification.** (**A**) Full trial showing the EEG trace (**top**) and rectified EEG trace (**bottom**) across pre-stimulation baseline (PRE, green), photic stimulation (STIM, magenta), and post-stimulation recovery (POST, orange). Dashed vertical lines indicate photic stimulation triggers, and shaded areas indicate identified bursts. (**B**) Magnified segment from the stimulation epoch. The arrow indicates a discrepancy between manual and automatic burst detection. The star indicates a trigger-induced visual evoked potential on the contralateral side that was not classified as a burst. This example illustrates the distinction between stimulus-locked evoked responses and burst events during burst suppression. P = probiotics and PA = perinatal asphyxia.

Each trial was intended to comprise exactly 60 s of stimulation-free baseline (PRE), 60 s of intermittent photic stimulation delivered at 0.5 Hz (STIM), and 60 s of stimulation-free recovery (POST) (Figure 1C), so that burst counts could be compared directly across epochs. For increased timing accuracy, epoch boundaries were measured relative to the actual photic stimulation signal recorded on the independent channel. This approach compensated for possible triggering drift between the stimulation command and the recorded stimulation events. A representative full trial with PRE, STIM, POST, trigger markers, and burst annotations is shown in Figure 2A.

A magnified stimulation epoch example that illustrates the distinction between a trigger-locked evoked response and a scored burst is shown in Figure 2B. Trials were excluded if signal quality was poor for reliable burst extraction, including major transient artifacts, loss of signal, baseline instability, or other marks that made the annotation impossible.

Three datasets (Reviewer 1, Reviewer 2, and Reviewer BURST) were generated using different approaches: Reviewer 1 manual burst counts constituted the primary dataset for burst count reactivity (BCR). To assess the reliability of this manual procedure, inter-rater agreement was evaluated on an independently scored subset of recordings performed by Reviewer 2. The Reviewer BURST dataset was generated by analyzing the entire valid recording dataset a second time, using the automated features.

### 2.8. Stimulation Trial Filtering

Reviewer 1 manual burst counts constituted the primary dataset for burst count analyses in the four experimental groups (C-PA, AB-PA, P-PA, and AB+P-PA). Pre-stimulation burst count (BC_Pre_) differed significantly between the groups (Appendix A, Table A1), as the depth of anesthesia was difficult to control during experiments. To standardize the depth of anesthesia, the allowable BC_Pre_ range was progressively narrowed until no significant differences remained between groups. This was achieved with BC_Pre_ limits of 8 to 15 bursts. All trials within this interval were retained. If an animal had no trial within the interval, the single trial with BC_Pre_ closest to the filter limits was retained so that all 45 animals remained represented after filtering. Under these criteria, the filtered Reviewer 1 dataset comprised 247 trials overall (75 C-PA, 57 AB-PA, 84 P-PA, and 31 AB+P-PA). After filtering, BC_Pre_ no longer differed between groups (one-way analysis of variance (ANOVA): F = 1.35, *p* = 0.259). This filtered Reviewer 1 dataset was used for all subsequent burst count analyses. The corresponding suppression-ratio-derived reactivity (BSRi) was subsequently used as an additional convergent metric, while ensuring that the SR_Pre_ >50%.

### 2.9. EEG Reactivity Analysis and Statistics

Within each group, BC_Pre_, burst count during stimulation (BC_Stim_), and burst count post stimulation (BC_Post_) were compared across epochs using repeated-measures ANOVA, followed by Holm-adjusted paired post hoc comparisons.

EEG reactivity was measured by analogy with previously described suppression-ratio-based indices during intermittent photic stimulation [16]. Burst count reactivity (BCR) was defined as the difference between burst count during stimulation (BC_Stim_) and burst count during the pre-stimulation baseline (BC_Pre_): BCR = BC_Stim_ − BC_Pre_. A larger BCR reflected greater burst occurrence during stimulation for the same BC_Pre_. The burst-suppression reactivity (BSR) was defined as the decrease in suppression ratio from the pre-stimulation baseline window to the stimulation window. Because SR depends on baseline suppression level and anesthetic depth, this change was normalized to the pre-stimulation value, yielding the burst-suppression reactivity index: BSRi = (SR_Pre_ − SR_Stim_)/SR_Pre_. Larger BSRi values, therefore, reflected a greater stimulation-induced reduction in suppression ratio and thus stronger EEG reactivity.

The reactivity measures were compared across groups at the trial level using one-way ANOVA with Tukey post hoc testing. Because the number of retained trials differed across animals, both BCR and BSRi were additionally aggregated as the median per rat, and group comparisons at the rat level were performed using pairwise Mann–Whitney U tests.

For methodological comparisons, receiver-operating characteristic analyses were restricted to the AB-PA versus C-PA comparison and were performed separately for BCR and BSRi at the trial level and, after median aggregation per animal, at the animal level. The area under the curve (AUC) was reported, with higher values of each metric favoring classification as C-PA.

## 3. Results

### 3.1. Manual Versus Automatic Burst Counts

**Figure 3:**
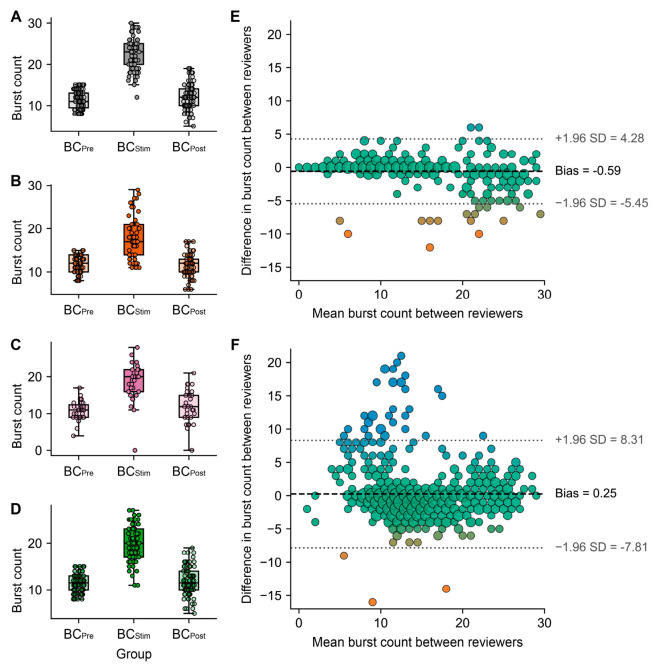
**Within-group burst count dynamics and agreement of burst counting methods assessed by Bland–Altman analysis.** Burst count distributions after BC_Pre_ filtering are shown for the four experimental groups: (**A**), C-PA; (**B**), AB-PA; (**C**), AB+P-PA; and (**D**), P-PA. For each group, burst counts are presented for the pre-stimulation baseline epoch (BC_Pre_), stimulation epoch (BC_Stim_), and post-stimulation recovery epoch (BC_Post_). In all groups, BC_Stim_ exceeded BC_Pre_, consistent with preserved photic reactivity during burst suppression. Box plots indicate the median and interquartile range, and points correspond to individual trials. Panel (**E**) compares paired burst counts from two manual reviewers across pooled BC_Pre_, BC_Stim_, and BC_Post_ observations (276 paired observations from 92 trials). Data points are represented with color ranging from blue (higher values) to orange (lower values). Panel (**F**) compares manual and automatic burst counts across pooled BC_Pre_, BC_Stim_, and BC_Post_ observations within the BC_Pre_ filtered dataset (699 paired observations from 233 trials). The x-axis represents the mean burst count of each pair, and the y-axis the difference between methods. Dashed horizontal lines indicate the mean bias, and dotted horizontal lines indicate the 95% limits of agreement. The same colors and color range used as in panel (**E**).

For downstream analysis, three burst count datasets were available: a primary manual dataset (Reviewer 1), an independently scored manual subset (Reviewer 2) used for inter-reviewer validation, and a reviewer-supervised automated dataset (Reviewer BURST). Agreement between the two manual reviewers was evaluated on 92 paired trials from six rats, yielding 276 paired BC_Pre_, BC_Stim_, and BC_Post_ observations pooled across epochs (Figure 3E). Mean pooled burst count was 14.71 for the primary manual counts and 15.30 for the second manual review. Bland–Altman analysis showed a small negative bias of −0.59 bursts for primary minus second manual counts, with 95% limits of agreement from −5.45 to 4.28 bursts. Absolute agreement was high, with an intraclass correlation coefficient ICC(A,1) of 0.943.

Within the BC_Pre_ filtered dataset, manual and automatic burst counts were compared in the subset of trials for which both measurements were available after filtering. This analysis included 233 matched trials from 43 rats, corresponding to 699 paired observations after pooling the pre-stimulation, stimulation, and post-stimulation epochs. The mean pooled burst count was 14.45 for manual counting and 14.20 for automatic counting. Bland–Altman analysis showed a small positive bias of 0.25 bursts for manual minus automatic counts, with 95% limits of agreement from −7.81 to 8.31 bursts. Absolute agreement was moderate to good, with an ICC(A,1) of 0.728 (Figure 3F). Overall, the automatic procedure reproduced the general pattern obtained by manual scoring with minimal systematic bias, although agreement was lower than between the two manual reviewers, and variability remained at the level of individual epoch counts.

### 3.2. Within-Group Burst Count Dynamics Across Epochs

After BC_Pre_ filtering, burst count varied significantly across BC_Pre_, BC_Stim_, and BC_Post_ in all four groups (Figure 3A–D): C-PA, F(2,148) = 316.53, *p* = 3.47 × 10^−54^; AB-PA, F(2,112) = 93.70, *p* = 1.22 × 10^−24^; AB+P-PA, F(2,60) = 65.38, *p* = 8.50 × 10^−16^; and P-PA, F(2,166) = 271.65, *p* = 4.47 × 10^−53^. Mean BC_Pre_, BC_Stim_, and BC_Post_ were 11.41, 22.33, and 11.79 in C-PA; 11.89, 18.14, and 11.56 in AB-PA; 10.94, 18.74, and 11.84 in AB+P-PA; and 11.50, 20.05, and 11.54 in P-PA. Holm-corrected post hoc testing showed that BC_Stim_ was significantly higher than both BC_Pre_ and BC_Post_ in every group (C-PA: *p* = 9.80 × 10^−33^ and *p* = 1.23 × 10^−28^; AB-PA: *p* = 4.43 × 10^−14^ and *p* = 1.50 × 10^−14^; AB+P-PA: *p* = 3.02 × 10^−10^ and *p* = 3.45 × 10^−9^; P-PA: *p* = 1.26 × 10^−32^ and *p* = 1.32 × 10^−29^). By contrast, BC_Pre_ and BC_Post_ did not differ significantly in any group (C-PA: *p* = 0.296; AB-PA: *p* = 0.341; AB+P-PA: *p* = 0.144; P-PA: *p* = 0.917). Thus, burst count remained clearly reactive to intermittent photic stimulation in all groups, with the largest stimulation-related increase in C-PA and smaller but still robust increases in AB-PA, AB+P-PA, and P-PA.

### 3.3. Group-Level Burst Count Reactivity (BCR)—Per Trial Analysis

**Figure 4:**
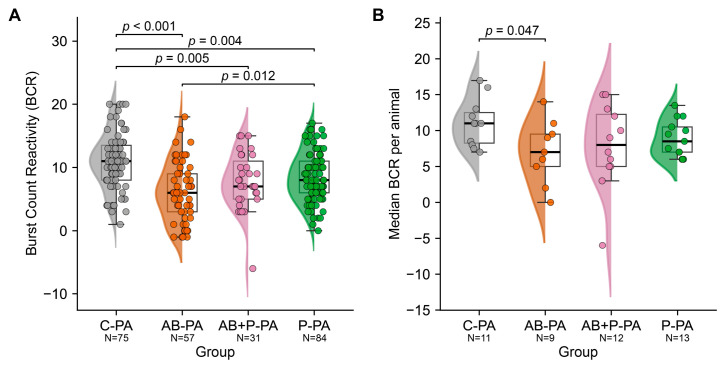
**Burst count reactivity across groups at the trial and animal level.** Panel (**A**): trial-level burst count reactivity (BCR = BC_Stim_ − BC_Pre_) after BC_Pre_ filtering in the four experimental groups. Panel (**B**): animal-level median BCR after aggregation of retained trials within each rat. Violin plots show the group distributions, box plots indicate the median and interquartile range, and individual points represent single trials (**left**) or single animals (**right**). Numbers below the group labels indicate the sample size. Significant pairwise comparisons are annotated on the plots. C = control; AB = antibiotics; P = probiotics; and PA = perinatal asphyxia.

As shown in Figure 4A, trial-level BCR differed significantly across groups after BC_Pre_ filtering (one-way ANOVA: F = 13.07, *p* = 6.06 × 10^−8^). Mean BCR was 10.92 ± 0.51 in C-PA (*n* = 75 trials), 8.55 ± 0.43 in P-PA (*n* = 84), 7.81 ± 0.81 in AB+P-PA (*n* = 31), and 6.25 ± 0.61 in AB-PA (*n* = 57). Tukey post hoc testing showed that AB-PA had significantly lower BCR than C-PA (mean difference 4.67, *p* < 0.001) and P-PA (mean difference 2.30, *p* = 0.012). C-PA also had significantly higher BCR than AB+P-PA (mean difference 3.11, *p* = 0.005) and P-PA (mean difference 2.37, *p* = 0.004). No significant difference was found between AB+P-PA and AB-PA (*p* = 0.374) or between AB+P-PA and P-PA (*p* = 0.849).

These findings indicate that antenatal maternal antibiotic exposure was associated with reduced burst count reactivity. Both groups that received probiotics showed higher BCR values than the AB-PA. This is consistent with attenuation of the antibiotic-associated reduction in reactivity. However, only P-PA differed significantly from AB-PA at the trial level, and neither probiotic-exposed group reached the control level observed in C-PA.

### 3.4. Group-Level Burst Count Reactivity (BCR)—Per Animal Analysis

As shown in Figure 4B, animal-level BCR aggregated as the median per rat was highest in C-PA and lowest in AB-PA. Group means ± SEM were 11.09 ± 1.00 in C-PA (*n* = 11 rats), 8.88 ± 0.71 in P-PA (*n* = 13), 7.83 ± 1.71 in AB+P-PA (*n* = 12), and 7.06 ± 1.46 in AB-PA (*n* = 9). Pairwise Mann–Whitney U testing showed that only the difference between C-PA and AB-PA remained significant at the animal level (U = 76.0, *p* = 0.0473). The other comparisons were not significant (C-PA vs. AB+P-PA, *p* = 0.1560; C-PA vs. P-PA, *p* = 0.0916; AB-PA vs. AB+P-PA, *p* = 0.6179; AB-PA vs. P-PA, *p* = 0.3143; AB+P-PA vs. P-PA, *p* = 0.6821).

Outliers were assessed within each group using the conventional 1.5 × interquartile range (IQR) criterion. This identified one low outlier in AB+P-PA (median BCR = −6), whereas no outliers were detected in C-PA, AB-PA, or P-PA. Visual inspection, therefore, suggests that the greater spread in AB+P-PA was driven in part by a single animal with paradoxically reduced bursting during stimulation. Despite this variability, the most robust animal-level contrast remained the reduction in BCR in AB-PA relative to C-PA, consistent with reduced EEG reactivity after antenatal maternal antibiotic exposure.

### 3.5. Reactivity Measurements by BSRi

**Figure 5:**
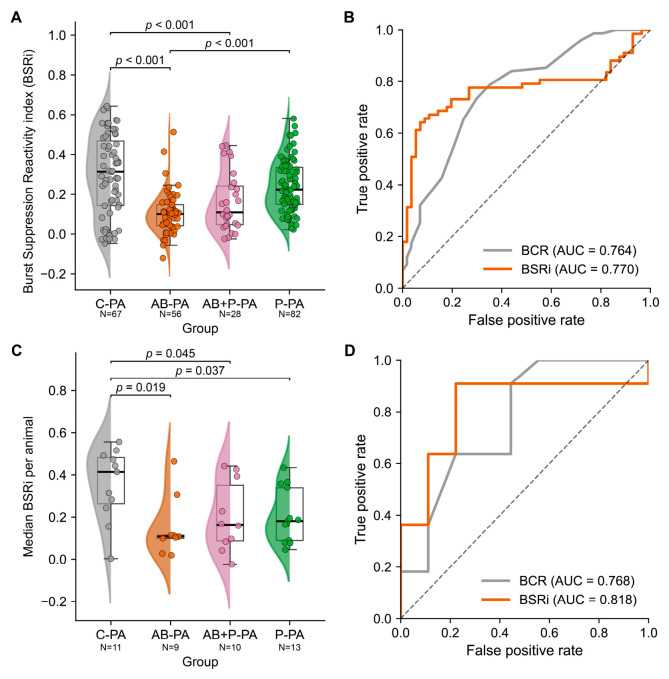
**Burst-suppression reactivity index across groups and ROC comparison with burst count reactivity.** Panels (**A**,**C**) show trial-level and animal-level BSRi after BC_Pre_ filtering. Panels (**B**,**D**) show ROC curves for AB-PA versus C-PA using BCR and BSRi at the trial and animal levels. The dashed diagonal line in the ROC plots indicates chance-level classification (AUC = 0.5). Violin plots show distributions, box plots indicate the median and interquartile range, and points indicate individual observations. Significant pairwise comparisons and AUC values are annotated on the plots.

On the same BC_Pre_ filtered dataset, BSRi also differed significantly across groups at the trial level and showed a similar overall ordering to BCR (Figure 5A). BSRi could be computed in 67 C-PA, 56 AB-PA, 28 AB+P-PA, and 82 P-PA trials. Mean BSRi was 0.296 ± 0.025 in C-PA, 0.240 ± 0.015 in P-PA, 0.163 ± 0.028 in AB+P-PA, and 0.104 ± 0.014 in AB-PA (one-way ANOVA: F = 18.04, *p* = 1.53 × 10^−10^). Tukey post hoc testing showed significantly lower BSRi in AB-PA than in C-PA (mean difference 0.192, *p* < 0.001) and P-PA (mean difference 0.135, *p* < 0.001), and significantly lower BSRi in AB+P-PA than in C-PA (mean difference 0.133, *p* = 0.0008). No significant difference was found between C-PA and P-PA (*p* = 0.113), between AB+P-PA and AB-PA (*p* = 0.342), or between AB+P-PA and P-PA (*p* = 0.101). At the animal level (Figure 5C), median BSRi per animal was also highest in C-PA. Group means ± SEM were 0.354 ± 0.052 in C-PA (*n* = 11), 0.206 ± 0.035 in P-PA (*n* = 13), 0.201 ± 0.053 in AB+P-PA (*n* = 10), and 0.150 ± 0.048 in AB-PA (*n* = 9). Pairwise Mann–Whitney testing showed higher median BSRi in C-PA than in AB-PA (U = 81.0, *p* = 0.0185), AB+P-PA (U = 84.0, *p* = 0.0448), and P-PA (U = 108.0, *p* = 0.0370), whereas no significant differences were detected among AB-PA, AB+P-PA, and P-PA. Overall, the BSRi analysis was consistent with the BCR results, showing reduced reactivity after gestational antibiotic exposure and attenuation of this reduction in the probiotic-exposed groups.

### 3.6. ROC Analysis for AB-PA Versus C-PA

Receiver-operating characteristic (ROC) analysis was performed on the BC_Pre_ filtered dataset to compare the ability of BCR and BSRi to distinguish AB-PA from C-PA (Figure 5B,D). At the trial level (Figure 5B), both metrics showed moderate discriminatory performance. BSRi yielded an AUC of 0.770 based on 123 trials, while BCR yielded an AUC of 0.764 based on 132 trials; in both cases, higher values favored classification as C-PA (Figure 5B). At the animal level, after median aggregation per animal (Figure 5D), the same ranking was preserved, with BSRi again outperforming BCR (AUC 0.818 vs. 0.768 across 20 animals). Thus, both EEG reactivity measures discriminated between antibiotic-exposed and control animals after perinatal asphyxia, with BSRi showing a small but consistent advantage over BCR in this AB-PA versus C-PA comparison.

## 4. Discussion

We investigated whether prolonged gestational antibiotic intake modifies the EEG reactivity in adult offspring in a rat model of HIBI after PA. The design was built upon our previous maternal microbiome disruption model and on our prior observation that EEG reactivity to photic stimulation under deep anesthesia detects functional impairment two months after PA. We found that the EEG reactivity of the adult offspring was lower after gestational antibiotic treatment than in controls. The magnitude of the effect was attenuated by probiotics coadministration.

### 4.1. Assessing EEG Reactivity During Burst Suppression: Rationale for an Event-Based Metric

Functional biomarkers are relevant because they can probe residual brain responsiveness and network integrity, adding to anatomical, histological, and behavioral endpoints. Among these biomarkers, the EEG-derived biomarkers are of particular importance because they can be used to capture large-scale cortical state dynamics in both clinical and research settings. In an attempt to reduce the complexity of the EEG signal, here we focused on the simple binary BS pattern induced by deep anesthesia [21,22,23].

The BS patterns are typically characterized by their SR. Nevertheless, SR is inherently unable to distinguish between burst occurrence and burst duration and remains sensitive to the anesthetic depth and agent. Modeling predicts that both burst duration and inter-burst interval are jointly determined by the metabolic and synaptic state, making SR sensitive to any factor that shifts the energetic balance of the cortical network [22]. To circumvent this limitation, previous studies focused on measuring changes in SR during stimulation and deriving metrics of BS reactivity normalized to anesthetic depth, such as BSRi [14,16,28].

To gain further mechanistic support for the SR changes, the current study was focused on burst counting as an event-based readout of stimulus-evoked burst recruitment [14,26,27]. The comparable ROC analysis for BSRi and BCR (Figure 5) suggests that the reactivity impairment was driven by changes in burst triggering rather than changes in burst duration.

**Figure A1:**
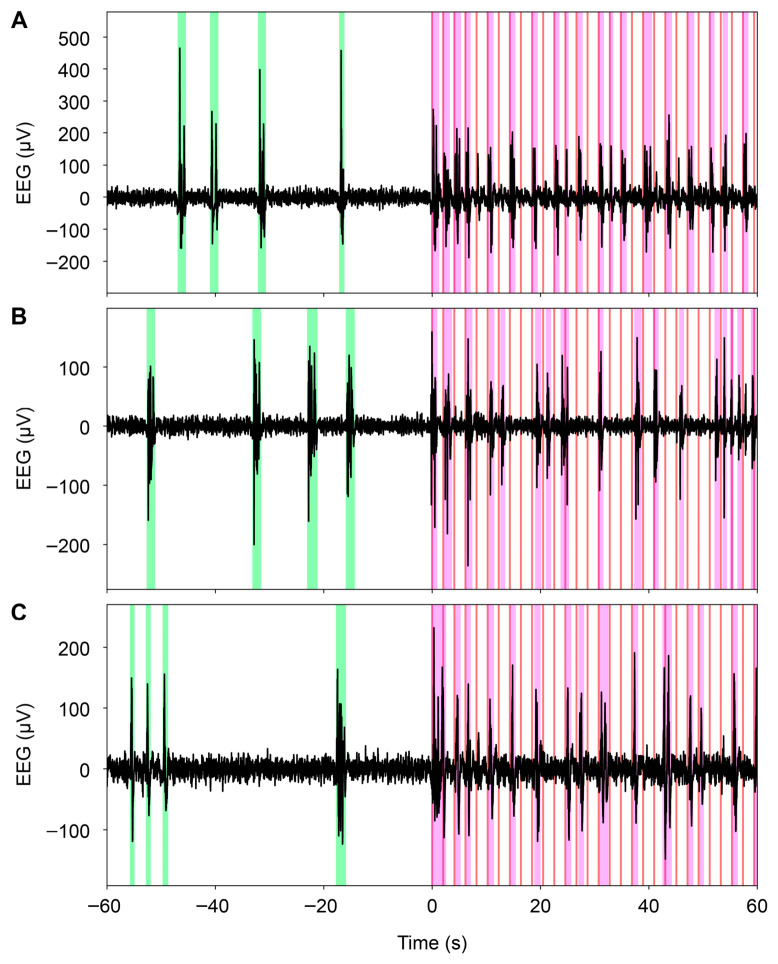
**Representative examples of burst-suppression reactivity in recordings with identical pre-stimulation burst count (BC_Pre_ = 4).** Representative EEG segments are shown for C-PA (**A**), AB-PA (**B**), and AB+P-PA (**C**). Only the pre-stimulation baseline (PRE) and photic stimulation (STIM) epochs are displayed. Vertical lines mark photic stimulation events, and shaded labels indicate visually identified bursts. BC_Pre_ = pre-stimulation burst count; C = control; AB = antibiotics; P = probiotics; and PA = perinatal asphyxia. Shaded labels are green for PRE and magenta for STIM.

It should be noted that BCR comparisons were limited to ensuring a similar BC_Pre_ between the groups. This anesthesia control could not be achieved during the experiments and required an offline strategy for BC_Pre_ filtering. We found that a range of 8 to 15 bursts ensured a comparable BC_Pre_ between the groups. The upper 15 burst limit resulted from the SR_Pre_ of 50%, which defines BS [21]. The eight-burst lower limit resulted from the fact that very deep anesthesia was not achieved in all experiments. Nevertheless, the filtered data showed the same qualitative tendency, including a stronger stimulation-related burst recruitment in the control group than in the antibiotic-exposed groups (Figure A1). Furthermore, the converging results between BCR and the automatic BSRi suggest that the filtering strategy did not confound the measured reactivity changes.

### 4.2. Burst Suppression Remains Reactive to Photic Stimulation After Perinatal Asphyxia

Across all treatment groups, burst-suppression patterns remained reactive to intermittent photic stimulation, with a consistent increase in BC and reduction in SR during stimulation as compared with baseline before stimulation. During the suppression phase, cortical networks accumulate a state of heightened readiness for transition, such that sensory input arriving during or near the end of a suppression period has an elevated probability of triggering a burst. The persistence of stimulus-evoked bursting is consistent with experimental evidence that the deeply discontinuous brain can retain stimulus sensitivity during BS [26,27] and with clinical findings that BS may remain reactive to photic stimulation in severe acquired brain injury [14]. In rodent HIBI, stimulation-evoked reactivity during BS has also been previously quantified using SR-based indices [16].

Photic burst recruitment depends on two linked but distinct steps: first, the sensory input must reach the cortex, and second, the cortical network must have the appropriate permissive state to allow the transition to a burst. In the context of the present study, preserved within-group reactivity is important because it indicates that between-group BCR differences reflect graded modulation of responsiveness rather than a binary presence or absence of reactivity. The antibiotic-associated impairment does not suggest that external stimulation fails to reach the brain or that the visual pathway is impaired, as indicated by the preservation of the visually evoked potential on the contralateral EEG channel (Figure 2B).

### 4.3. Gestational Antibiotic Exposure Is Associated with Reduced Adult EEG Reactivity After Perinatal Asphyxia

Prolonged antibiotic administration during gestation has been shown in related work to modify maternal microbiome structure and to affect immune, metabolic, and inflammatory pathways that are relevant to fetal brain development and later injury response [8,31,32]. Additionally, the gut microbiome can influence neuroinflammatory response and lesion extent after neonatal HIBI, hinting at a mechanistic bridge between the gut ecology and HIBI susceptibility [38].

At both the trial level and at the rat level, gestational antibiotic exposure was associated with reduced BCR and BSRi after PA. Because reduced BS reactivity has been associated with greater functional impairment after HIBI [16], the reduced reactivity is consistent with a greater long-term functional burden of PA-related HIBI in antibiotic-exposed animals.

Consistent with our previous studies on the same model under isoflurane anesthesia [16], we found that when the reactivity was decreased under chloral hydrate, bursts still followed photic stimulation events, while an increasing number of stimuli did not trigger bursts (Figure A1), suggesting a transient refractoriness or the gating of burst recruitment [39]. Although assessing the changes in stimulus refractoriness during BS was beyond the scope of this study, it is tempting to suspect that they reflected a metabolic insufficiency [40]. The impaired reactivity could also point towards a metabolic limit that is reached [22]. In this scenario, the sensory input may still evoke a time-locked cortical response, but the network could remain below the threshold required for time-locked burst propagation [22,39]. Addressing this could require advanced connectivity analysis, including phase amplitude coupling and related Bayesian inference frameworks to determine whether the impaired reactivity primarily indicates a disrupted sensory transmission, a modified network excitability, or an altered large-scale integration [41].

### 4.4. Probiotic Exposure Is Consistent with Attenuation of the Antibiotic Effect Without Full Normalization

Although no direct microbiome profiling was performed in the present cohort, prior model-development work using a comparable gestational antibiotic paradigm demonstrated maternal gut microbiome disruption and altered early offspring outcomes after PA [9]. Rather than repeating these studies to explore whether the impairment of reactivity after antibiotic administration was mediated by microbiome disruption, we tested the potentially modulatory effect of probiotic coadministration [17].

Across analyses, probiotic exposure was associated with attenuation of the antibiotic-related reduction in both BCR and BSRi, supporting microbial involvement in the observed effects. Nevertheless, at the trial level, the reactivity after probiotics remained reduced as compared to controls, consistent with a partial attenuation. This was not surprising because the probiotic intervention was not designed as a full antidote to antibiotic exposure. No dose-finding studies were performed, probiotic strain persistence was not confirmed, and the absolute amount of active compound delivered through ad libitum drinking water may have varied between animals. Probiotic supplementation, therefore, may not have fully prevented antibiotic-driven dysbiosis, and the gestational exposure window may have limited efficacy [8].

It should also be mentioned that gestational antibiotic exposure alters the offspring’s gut microbiota directly [42], and offspring microbiota composition can modulate the severity of hypoxic–ischemic brain injury [38]. Probiotics could therefore mitigate antibiotic-induced dysbiosis [17], providing a biologically plausible framework for the attenuation pattern observed here. Future studies that incorporate microbiome profiling, confirmation of probiotic strain persistence, controlled probiotic dosing, and mechanistic immune or metabolic readouts will be needed to determine whether the observed attenuation reflects a biologically meaningful rescue process or sampling variability.

### 4.5. Limitations and Future Directions

By design, the study was limited to a single functional neurophysiological readout, at a single time point. The EEG reactivity during burst suppression was chosen to build upon previous studies on the same model, where burst-suppression reactivity to photic stimulation detected impairment two months after PA [16]. This limited the ability to characterize the developmental trajectory. It remains unknown whether the phenotype emerges early on after PA or if it reflects a failed recovery during maturation. Furthermore, EEG reactivity measures provide information on large-scale cortical network dysfunction, but cannot inform about lesion volumes, inflammation, cellular and molecular injury, scarring, and cognitive outcome. Multimodal outcome measures are required to gain mechanistic insight into the factors underlying the impairment of reactivity [43].

The study exposed all offspring to the PA procedure. Our previous study [16] did show that PA alone can impair the BS reactivity as compared to normoxic rats; the magnitude of BSRi in our current C-PA group is comparable to the previously reported changes after PA. Taken together, it is reasonable to suspect that the observed further reactivity impairment by gestational antibiotic exposure occurred through aggravation of the severity of HIBI. Nevertheless, our previous studies were carried out under a different anesthetic regimen and only on male rats, and such factors could affect the magnitude of the reactivity changes [16,29,44,45]. Further research should be carried out to confirm that the antibiotics and probiotics treatment used in this study do not significantly alter the reactivity changes under normoxic conditions in this model.

In this study, a complete burst count was performed manually by a single trained investigator in a blinded workflow. Although inter-rater agreement on an independently scored subset supported the reliability of this approach, manual annotation remains labor-intensive and limits throughput relative to automated pipelines. An additional advantage of the dataset generated is that it could serve as a training resource for automated burst classifiers in future studies, improving throughput and reproducibility across laboratories.

Reactivity measures in BS, like BCR and BSRi, are potentially translational EEG biomarkers of HIBI. In humans, intracranial recordings have shown that BS is not spatially homogeneous. Bursts can remain localized, and the burst onset can vary across the cortical regions, with delays of hundreds of milliseconds [46]. Thus, electrode placement, geometry, impedance, and local signal properties [46,47] could affect the absolute BS reactivity values. To minimize this confounder, here we focused on a whole-brain reduced montage aimed to quantify global reactivity. However, we do not suggest that clinical patients should be induced to burst suppression in order to assess BS reactivity. In continuous EEG backgrounds, other quantitative reactivity approaches, such as default EEG macrostate reactivity (DER), may be more suitable to assess HIBI, even with a reduced montage [15]. Such reactivity methods should be considered in future clinical trials following birth asphyxia.

## 5. Conclusions

Our data suggest that prolonged gestational antibiotic exposure increased the severity of perinatal asphyxia as measured by EEG reactivity in adult offspring. The converging BCR and BSRi measures support burst-suppression reactivity as a functional neurophysiological readout in this PA model and support further methodological development of EEG reactivity measures for translational studies of hypoxic–ischemic brain injury.

## Data Availability

The data presented in this study are available from the corresponding author upon reasonable request.

## Abbreviations

The following abbreviations are used in this manuscript:

AB	Antibiotic
AB+P	Antibiotic plus probiotic
ACNS	American Clinical Neurophysiology Society
ANOVA	Analysis of variance
BC	Burst count
BC_Post_	Burst count during recovery (post-stimulation)
BC_Pre_	Burst count at baseline (pre-stimulation)
BC_Stim_	Burst count during stimulation
BCR	Burst count reactivity
BS	Burst suppression
BSRi	Burst-suppression reactivity index
C	Control
CFU	Colony-forming units
ECG	Electrocardiogram
EEG	Electroencephalogram
AI	Generative artificial intelligence
HDF5	Hierarchical Data Format version 5
HIBI	Hypoxic–ischemic brain injury
ICC	Intraclass correlation coefficient
IQR	Interquartile range
P	Probiotic
PA	Perinatal asphyxia
POST	Post-stimulation epoch
PRE	Pre-stimulation epoch
SD	Standard deviation
SEM	Standard error of the mean
SR	Suppression ratio
SR_Pre_	Suppression ratio at baseline
SR_Stim_	Suppression ratio during stimulation
STIM	Stimulation epoch

## Appendix A

**Table A1:** **Reviewer 1 pre-stimulation burst count (BC_Pre_) before application of BC_Pre_ filter**. (A) Descriptive statistics. (B) One-way ANOVA summary. (C) Tukey post hoc comparisons.

**(A)**													
**Group**		**Mean**		**Median**			**SD**		**N**			**SEM**	
C-PA		11.62		12.00			4.31		121			0.39	
AB-PA		10.01		11.00			3.71		84			0.40	
AB+P-PA		7.99		7.00			4.86		74			0.56	
P-PA		10.80		11.00			3.92		122			0.35	
**(B)**													
**Test**			**F**			***p*** **Value**				**Groups Tested**			
One-way ANOVA			12.24			1.12 × 10^−7^				4			
**(C)**													
**Group 1**	**Group 2**		**Mean Diff**		**Adj. *p***			**Lower**			**Upper**		**Sig.**
C-PA	P-PA		−0.82		0.4259			−2.20			0.57		No
AB-PA	C-PA		1.61		0.0357			0.07			3.14		Yes
AB-PA	P-PA		0.79		0.5419			−0.74			2.32		No
AB+P-PA	C-PA		3.63		0.00			2.04			5.23		Yes
AB+P-PA	AB-PA		2.03		0.0136			0.30			3.75		Yes
AB+P-PA	P-PA		2.82		0.00			1.23			4.41		Yes

Analysis of Reviewer 1 pre-stimulation burst count (BC_Pre_) before application of the BC_Pre_ filter. Panel (A) shows descriptive statistics, panel (B) the one-way ANOVA summary, and panel (C) the Tukey post hoc comparisons. Descriptive statistics are shown as mean, median, standard deviation (SD), number of trials (N), and standard error of the mean (SEM). Adjusted *p*-values are reported for the post hoc analysis. AB = antibiotics; P = probiotics; C = control; and PA = perinatal asphyxia.

### The Impaired Reactivity Is Also Present in the Pre-Filtered Dataset

Before BC_Pre_ filtering, Reviewer 1 pre-stimulation burst count differed across groups, with the lowest baseline burst count in AB+P-PA and intermediate values in AB-PA relative to C-PA and P-PA (Table A1). These differences confirm that the unfiltered dataset contained between-group variation in baseline burst-suppression depth, which justified the use of BC_Pre_ filtering in the primary burst count analysis.

At the same time, the appendix figure indicates that the reactivity phenotype is not explained solely by this baseline imbalance. In representative recordings matched for pre-stimulation burst count (BC_Pre_ = 4), the stimulation epoch is accompanied by more robust burst recruitment in C-PA (Figure A1A) than in the antibiotic-exposed examples (Figure A1B,C), consistent with weaker EEG reactivity in the latter. Thus, these results support the interpretation that impaired burst-suppression reactivity is already visible qualitatively in the unfiltered material, while the filtered analysis remains the appropriate primary comparison because it controls baseline burst-suppression differences across groups.

## References

[1] Herrera-Marschitz M., Golubnitchaja O., Bustamante D., Morales P., Klawitter V., Fiedler J.L., Morelli M., Tasker A., Gomez-Urquijo S., Hökfelt T. (2007). Perinatal asphyxia as the leading cause of death and brain injury of newborns: Prognosis and neuroprotection of long-term outcomes. GMS J. Med. Educ..

[2] Vannucci R.C., Vannucci S.J. (1997). A model of perinatal hypoxic-ischemic brain damage. Ann. N. Y. Acad. Sci..

[3] Northington F.J. (2006). Brief update on animal models of hypoxic-ischemic encephalopathy and neonatal stroke. ILAR J..

[4] Lu J., Lu L., Yu Y., Baranowski J., Claud E.C. (2020). Maternal administration of probiotics promotes brain development and protects offspring’s brain from postnatal inflammatory insults in C57/BL6J mice. Sci. Rep..

[5] Isac S., Panaitescu A.M., Spataru A., Iesanu M., Totan A., Udriste A., Cucu N., Peltecu G., Zagrean L., Zagrean A.-M. (2017). Trans-resveratrol enriched maternal diet protects the immature hippocampus from perinatal asphyxia in rats. Neurosci. Lett..

[6] Isac S., Panaitescu A.M., Iesanu M., Grigoras I.F., Totan A., Udriste A., Cucu N., Peltecu G., Zagrean L., Zagrean A.-M. (2018). Maternal high-fat diet modifies the immature hippocampus vulnerability to perinatal asphyxia in rats. Neonatology.

[7] Isac S., Panaitescu A.M., Iesanu M.I., Zeca V., Cucu N., Zagrean L., Peltecu G., Zagrean A.-M. (2020). Maternal citicoline-supplemented diet improves the response of the immature hippocampus to perinatal asphyxia in rats. Neonatology.

[8] Morozan V.-P., Ionescu M.I., Zahiu C.M.D., Catrina A.M., Racoviță A., Chirilă A.-T., Dogaru I.-A., Ciotei C., Pircalabioru G.G., Zăgrean A.-M. (2025). Does the maternal gut microbiome influence the outcome of perinatal asphyxia?. Antioxidants.

[9] Ionescu M.I., Maria Catrina A., Dogaru I.A., Catalina Barbalata D., Ciotei C., Haidoiu C., Suhaianu V., Pircalabioru G.G., O’mAhony S.M., Zagrean A.-M. (2024). MICROBIOME: The trials and errors of developing an experimental model to study the impact of maternal gut microbiome disruption on perinatal asphyxia. Reprod. Fertil..

[10] Panaitescu A.M., Isac S., Pavel B., Ilie A.S., Ceanga M., Totan A., Zagrean L., Peltecu G., Zagrean A.M. (2018). Oxytocin reduces seizure burden and hippocampal injury in a rat model of perinatal asphyxia. Acta Endocrinol..

[11] Kharoshankaya L., Stevenson N.J., Livingstone V., Murray D.M., Murphy B.P., Ahearne C.E., Boylan G.B. (2016). Seizure burden and neurodevelopmental outcome in neonates with hypoxic-ischemic encephalopathy. Dev. Med. Child Neurol..

[12] Trowbridge S.K., Condie L.O., Landers J.R., Bergin A.M., Grant P.E., Krishnamoorthy K., Rofeberg V., Wypij D., Staley K.J., Soul J.S. (2023). Effect of neonatal seizure burden and etiology on the long-term outcome: Data from a randomized, controlled trial. Ann. Child. Neurol. Soc..

[13] Cornet M.-C., Numis A.L., Wusthoff C.J., Bernardo D., Mietzsch U., Thomas C., Natarajan N., Ahmad K.A., Scheffler A., Juul S.E. (2025). Automated EEG background analysis and 2-year outcomes in neonatal hypoxic-ischemic encephalopathy. JAMA Netw. Open.

[14] Nita D.A., Moldovan M., Sharma R., Avramescu S., Otsubo H., Hahn C.D. (2016). Burst-suppression is reactive to photic stimulation in comatose children with acquired brain injury. Clin. Neurophysiol..

[15] Serban C.-A., Barborica A., Roceanu A.-M., Mindruta I., Ciurea J., Pâslaru A.C., Zăgrean A.-M., Zăgrean L., Moldovan M. (2022). A method to assess the default EEG macrostate and its reactivity to stimulation. Clin. Neurophysiol..

[16] Pâslaru A.-C., Călin A., Morozan V.-P., Stancu M., Tofan L., Panaitescu A.M., Zăgrean A.-M., Zăgrean L., Moldovan M. (2024). Burst-suppression EEG reactivity to photic stimulation—A translational biomarker in hypoxic–ischemic brain injury. Biomolecules.

[17] Łukasik J., Dierikx T., Johnston B.C., de Meij T., Szajewska H. (2024). Systematic review: Effect of probiotics on antibiotic-induced microbiome disruption. Benef. Microbes.

[18] Latif A., Shehzad A., Niazi S., Zahid A., Ashraf W., Iqbal M.W., Rehman A., Riaz T., Aadil R.M., Khan I.M. (2023). Probiotics: Mechanism of action, health benefits and their application in food industries. Front. Microbiol..

[19] Jarde A., Lewis-Mikhael A.-M., Moayyedi P., Stearns J.C., Collins S.M., Beyene J., McDonald S.D. (2018). Pregnancy outcomes in women taking probiotics or prebiotics: A systematic review and meta-analysis. BMC Pregnancy Childbirth.

[20] Chen Y., Li Z., Tye K.D., Luo H., Tang X., Liao Y., Wang D., Zhou J., Yang P., Li Y. (2019). Probiotic supplementation during human pregnancy affects the gut Microbiota and immune status. Front. Cell. Infect. Microbiol..

[21] Hirsch L.J., Fong M.W.K., Leitinger M., LaRoche S.M., Beniczky S., Abend N.S., Lee J.W., Wusthoff C.J., Hahn C.D., Westover M.B. (2021). American clinical neurophysiology society’s standardized critical care EEG terminology: 2021 version. J. Clin. Neurophysiol..

[22] Ching S., Purdon P.L., Vijayan S., Kopell N.J., Brown E.N. (2012). A neurophysiological-metabolic model for burst suppression. Proc. Natl. Acad. Sci. USA.

[23] Shanker A., Abel J.H., Schamberg G., Brown E.N. (2021). Etiology of burst suppression EEG patterns. Front. Psychol..

[24] Westover M.B., Kim S.-E., Ching S., Purdon P.L., Brown E.N. (2015). Robust control of burst suppression for medical coma. J. Neural Eng..

[25] Pawar N., Barreto Chang O.L. (2021). Burst Suppression During General Anesthesia and Postoperative Outcomes: Mini Review. Front. Syst. Neurosci..

[26] Kroeger D., Amzica F. (2007). Hypersensitivity of the anesthesia-induced comatose brain. J. Neurosci..

[27] Hudetz A.G., Imas O.A. (2007). Burst activation of the cerebral cortex by flash stimuli during isoflurane anesthesia in rats. Anesthesiology.

[28] Călin A., Kumaraswamy V.M., Braver D., Nair D.G., Moldovan M., Simon M.V. (2014). Intraoperative somatosensory evoked potential monitoring decreases EEG burst suppression ratio during deep general anesthesia. J. Clin. Neurophysiol..

[29] Parodi G., Leite G., Pimentel M.L., Barlow G.M., Fiorentino A., Morales W., Weitsman S., Mathur R. (2022). The response of the rodent gut microbiome to broad-spectrum antibiotics is different in males and females. Front. Microbiol..

[30] Semple B.D., Blomgren K., Gimlin K., Ferriero D.M., Noble-Haeusslein L.J. (2013). Brain development in rodents and humans: Identifying benchmarks of maturation and vulnerability to injury across species. Prog. Neurobiol..

[31] Fröhlich E.E., Farzi A., Mayerhofer R., Reichmann F., Jačan A., Wagner B., Zinser E., Bordag N., Magnes C., Fröhlich E. (2016). Cognitive impairment by antibiotic-induced gut dysbiosis: Analysis of gut microbiota-brain communication. Brain Behav. Immun..

[32] O’Connor R., Moloney G.M., Fulling C., O’Riordan K.J., Fitzgerald P., Bastiaanssen T.F.S., Schellekens H., Dinan T.G., Cryan J.F. (2021). Maternal antibiotic administration during a critical developmental window has enduring neurobehavioural effects in offspring mice. Behav. Brain Res..

[33] Deng H., Yu Y., Sha Q., Sun W., Liang L., Ren F., Ji H., Shen X., Fan X. (2023). Construction of antibiotic-induced depression mice model and the function of intestinal microbiota. Front. Microbiol..

[34] Faas M.M., Liu Y., Wekema L., Weiss G.A., van Loo-Bouwman C.A., Silva Lagos L. (2023). The Effect of Antibiotics Treatment on the Maternal Immune Response and Gut Microbiome in Pregnant and Non-Pregnant Mice. Nutrients.

[35] Leclercq S., Mian F.M., Stanisz A.M., Bindels L.B., Cambier E., Ben-Amram H., Koren O., Forsythe P., Bienenstock J. (2017). Low-dose penicillin in early life induces long-term changes in murine gut microbiota, brain cytokines and behavior. Nat. Commun..

[36] Nagate T., Chino T., Nishiyama C., Okuhara D., Tahara T., Maruyama Y., Kasahara H., Takashima K., Kobayashi S., Motokawa Y. (2007). Diluted isoflurane as a suitable alternative for diethyl ether for rat anaesthesia in regular toxicology studies. J. Vet. Med. Sci..

[37] Ilie A., Ciocan D., Zagrean A.-M., Nita D.A., Zagrean L., Moldovan M. (2006). Endogenous activation of adenosine A(1) receptors accelerates ischemic suppression of spontaneous electrocortical activity. J. Neurophysiol..

[38] Drobyshevsky A., Synowiec S., Goussakov I., Fabres R., Lu J., Caplan M. (2024). Intestinal microbiota modulates neuroinflammatory response and brain injury after neonatal hypoxia-ischemia. Gut Microbes.

[39] Amzica F. (2009). Basic physiology of burst-suppression. Epilepsia.

[40] Moldovan M., Constantinescu A.O., Balseanu A., Oprescu N., Zagrean L., Popa-Wagner A. (2010). Sleep deprivation attenuates experimental stroke severity in rats. Exp. Neurol..

[41] Castillo-Barnes D., Ortiz A., Figueiredo P., Gallego-Molina N.J. (2025). A Bayesian framework for phase-amplitude cross-frequency coupling inference: Application to reading disability detection. Expert. Syst. Appl..

[42] Madany A.M., Hughes H.K., Ashwood P. (2022). Antibiotic treatment during pregnancy alters offspring gut Microbiota in a sex-dependent manner. Biomedicines.

[43] Azabou E., Fischer C., Guerit J.M., Annane D., Mauguiere F., Lofaso F., Sharshar T. (2017). Neurophysiological assessment of brain dysfunction in critically ill patients: An update. Neurol. Sci..

[44] Kenny J.D., Westover M.B., Ching S., Brown E.N., Solt K. (2014). Propofol and sevoflurane induce distinct burst suppression patterns in rats. Front. Syst. Neurosci..

[45] Fleischmann A., Pilge S., Kiel T., Kratzer S., Schneider G., Kreuzer M. (2018). Substance-specific differences in human electroencephalographic burst suppression patterns. Front. Hum. Neurosci..

[46] Lewis L.D., Ching S., Weiner V.S., Peterfreund R.A., Eskandar E.N., Cash S.S., Brown E.N., Purdon P.L. (2013). Local cortical dynamics of burst suppression in the anaesthetized brain. Brain.

[47] Moldovan M., Calin A., Kumaraswamy V.M., Braver D., Simon M.V. (2016). Burst-Suppression Ratio on Electrocorticography Depends on Interelectrode Distance. J. Clin. Neurophysiol..

